# Oral health-related quality of life in patients infected with HIV, Iran: a cross-sectional study

**DOI:** 10.1186/s12903-021-01660-7

**Published:** 2021-06-16

**Authors:** S. Shaghaghian, E. Saranjam, M. Homayooni

**Affiliations:** 1 Communicable Diseases Unit, Shohadae-Enghelab Health Center, Shiraz University of Medical Sciences, P. O. Box: 7164788363, Shiraz, Iran; 2 Shiraz HIV/AIDS Research Center, Institute of Health, Shiraz University of Medical Sciences, Shiraz, Iran; 3 School of Nursing and Midwifery, Shiraz University of Medical Sciences, Shiraz, Iran; 4 Voluntary Counseling and Testing Center, Shiraz University of Medical Science, Shiraz, Iran

**Keywords:** Human Immunodeficiency Virus, Oral health, Quality of life

## Abstract

**Background:**

The life expectancy of patients with Human Immunodeficiency Virus (HIV) has increased, but its oral manifestations can affect the Oral Health-Related Quality of Life (OHRQoL) of these patients. This study aimed to evaluate OHRQoL in HIV-infected patients and determine its related factors.

**Methods:**

In this cross-sectional study, 250 HIV-infected patients were randomly selected from Shiraz Voluntary Counseling and Testing center in 2019. OHRQoL was measured using the revised Geriatric Oral Health Assessment Index for HIV patients. The associations between patients’ OHRQoL and demographic characteristics were examined.

**Results:**

The mean score of the patients' OHRQoL was 24.55 ± 6.27. The lowest and highest scores belonged to the psychosocial and pain categories, respectively. In the univariate analysis, the OHRQoL was significantly associated with patients' age (*p* = 0.012), duration of the disease (*p* = 0.009), job (*p* = 0.006), edentulous status (*p* = 0.003), and wearing denture (*p* < 0.001). However, in the multiple linear regression analysis, a significant difference was found only between denture wearing and non-denture wearing patients (*p* ≤ 0.001).

**Conclusions:**

The OHRQoL of HIV-infected patients was not optimal since most of the patients were worried about their oral and dental health problems. The OHRQoL was significantly better in denture-wearing patients. This finding highlights the impact of unmet dental needs on OHRQoL in HIV-infected patients. Therefore, dental services coverage for patients with HIV is essential, so that they can have timely access to oral health care. Furthermore, dentures should be considered as an unmet healthcare need among HIV patients in public health policies.

## Background

Quality of life is individuals’ understanding of their living conditions in the cultural context and value system in which they live [1]. Health-related quality of life (HRQoL) consists of those aspects of quality of life that are influenced by the presence of disease or treatment [1]. Oral health-related quality of life (OHRQoL) is defined as the impact of oral conditions on people's everyday life [2]. World Health Organization recognizes this multidimensional concept as an important part of general and oral health and identifies it as a significant part of the Global Oral Health Program [3].

Health problems can impair human wellbeing and negatively affect patients' quality of life [4]. Therefore, to assess the overall impact of chronic diseases on patients, researchers use HRQoL measures. Infection with Human Immunodeficiency Virus (HIV) is recognized as one of the most important public health problems in the world [4]. However, because of the modern therapeutic measures, the life expectancy of the patients infected with HIV has increased. Nevertheless, because of several health-related outcomes, which lead to chronic comorbidities, HRQoL in HIV-infected patients has been reported as poor [5]. In addition, many of the patients suffer from oral health problems and experience at least one oral disease during their illness [6]. The oral manifestations of HIV can compromise some of the important day-to-day activities such as the ability to chew and swallow food comfortably, speak, and interact socially [7]. Therefore, HIV infections can affect OHRQoL [6, 8].

To assess OHRQoL, several questionnaires have been developed since 1980, many of which have focused on the elderly. One of them was Geriatric Oral Health Assessment Index (GOHAI), a self-reported measure originally designed for older adults. In 2002, Coulter and coworkers modified GOHAI to use it for assessing the OHRQoL in patients with HIV. They removed the items specific to the elderly and added items relevant to HIV disease [7].

Several studies have evaluated the OHRQoL in HIV-infected patients. In a study conducted in Malaysia, the impact of oral diseases was greater in HIV-infected patients than that in the general population [6]. The greatest impact was the discomfort due to food being stuck and difficulty in chewing food [6]. A study conducted in Brazil also showed a high prevalence of frequent/very frequent impact on the OHRQoL in children and adolescents with HIV [8]. In a study conducted in the United States, the OHRQoL in HIV-infected women was 10% poorer than that in HIV-uninfected women [9]. Parish et al. [10] also showed a strong positive association between poor OHRQoL and unmet dental needs in women living with HIV. They recommended that the dentists should consider OHRQoL evaluation in the management of the patients to detect those who had significant oral health impacts [10].

In Iran, all patients infected with HIV are referred to the Voluntary Counseling and Testing (VCT) center to receive the necessary services such as those related to dental care. To provide services well matched with the patients' needs, authorities require information about the patients' OHRQoL. Therefore, this study was conducted to evaluate the OHRQoL in patients infected with HIV and to determine its related factors.

## Methods

### Participants

The target population for this cross-sectional study was HIV-infected patients who were registered in the VCT center affiliated to Shiraz University of Medical Sciences in 2019. Patients younger than 18 years, those who did not consent to participate in the study or were uncooperative, those who were in prison, and those for whom no telephone number was recorded were excluded from the study.

### Sample size calculation and sampling

To accurately determine the sample size, we conducted a pilot study because we did not find a similar previous study in the Iranian population. In this cross-sectional study, the OHRQoL was determined in 18 HIV-infected patients. Using the sample size formula for determining the mean of a population and considering the mean and standard deviation of the obtained OHRQoL in the pilot study, α = 0.05, and d = 1, a statistician calculated the sample size as 163 patients. However, the sample size was increased to 250 to be adequate for achieving other objectives of the study and to compensate for possible withdrawals from the study. Therefore, a random sample of 250 patients was considered representative of the target population. A patient who was registered in Shiraz VCT center had a code number that was recorded in Iran national software for control of HIV/AIDS. Entering the codes in SPSS software, we selected 250 patients by simple random sampling. However, only 219 (87.6%) patients participated in the study. Fifteen uncooperative and three under 18-year-old patients were excluded from the study. As eight selected patients did not fill out their questionnaires completely, we did not enter their data. Furthermore, five patients were excluded because no telephone number was recorded for them in the VCT center.

### The questionnaire

To evaluate the OHRQoL in the selected patients, we used the modified version of the Geriatric Oral Health Assessment Index that was revised for the patients infected with HIV [7]. The questionnaire consisted of seven questions in four categories: oral and psychosocial functions, pain, and social activity. Two questions evaluated each of the first three categories and one question assessed how much the patients' oral health interfered with their social activities (Table 1).

**Table 1:** Participants' oral health-related quality of life (N = 219)

Category	Questions	Scores (Mean ± SD)	Standardized Scores (Mean ± SD)
Pain	In the last 4 weeks, how often did you have pain or discomfort in your mouth?	4.03 ± 1.24	80.64 ± 24.78
Pain	In the last 4 weeks, how often did you use medication to relieve pain or discomfort in your mouth?	4.33 ± 1.06	86.67 ± 21.19
Function	In the last 4 weeks, how often were you able to swallow comfortably?	3.44 ± 1.06	68.76 ± 25.19
Function	In the last 4 weeks, how often did you limit the kinds or amounts of foods you ate because of problems in your mouth?	3.67 ± 1.32	73.33 ± 26.50
Social	In the last 4 weeks, how often did your oral health interfere with your social activities?	4.17 ± 1.18	83.47 ± 23.59
Psychosocial	In the last 4 weeks, how often were you worried or concerned about problems in your mouth?	2.40 ± 1.50	48.13 ± 30.07
Psychosocial	In the last 4 weeks, how often were you pleased or happy with the look of your mouth?	2.50 ± 1.58	50.04 ± 31.67
Overall oral health-related quality of life		24.55 ± 6.27	70.15 ± 17.90

SD: Standard Deviation

The response options for the questions were a five-point scale including all, most, some, a little, and none of the time. Each question was scored from 1 to 5. Summing up the scores of the questions, we scored the questionnaires from 7 to 35; the higher the score, the better the OHRQoL. To compare the score of the overall OHRQoL with that of each question, we standardized all scores from 0 to 100%.

The reliability of the English version of the questionnaire had been confirmed in a previous study [7]. However, we translated the questionnaire into the Persian language and evaluated the reliability and validity of the Persian version as follows:

- Reliability: Conducting a pilot study and using Cronbach's α, we confirmed its reliability (α = 0.83).
- Qualitative face validity: Fifteen faculty members of Shiraz University of Medical Sciences were asked to assess the sentences used in each question regarding the difficulty level, appropriateness, ambiguity, and relevance to the main purpose. Using their opinions, we edited the problematic sentences. Then the faculty members confirmed the face validity of the questionnaire.
- Quantitative face validity: We interviewed 18 HIV-infected patients to score the importance of each question. The face validity was determined by using the item impact method. In this method, the importance was scored from 1 (not important at all) to 5 (completely important). Impact scores of the questions were measured by using the following formula:

Impact score=percentage of the participants who gave each item scores 4 or 5 × means of importance score for each question.

An impact score of ≥1.5 was considered appropriate [11]. In the present study, all the questions were scored ≥1.5; therefore, no question was deleted.

- Qualitative content validity: Eighteen experts in dentistry and nursing were asked to express their opinions about the questionnaire regarding the grammar, use of proper words, and placement of the items in the proper place. Then the questionnaire was corrected according to the experts' opinions and they confirmed its content validity.

### Data collection

Our gatekeeper was one of the personnel of the VCT center that called the randomly selected patients. She provided them with adequate information about the study and made an appointment for those who were willing to participate in the study. In the scheduled appointment, the staff completed the questionnaires through an interview with all the referred patients. She also recorded the participants’ demographic and health characteristics either by asking the patients directly or by searching the patients' records.

### Statistical methods

The collected data were analyzed using SPSS software version 20 (SPSS Inc., Chicago, Illinois, USA). To evaluate the association between the patients' OHRQoL and their characteristics, we used Pearson correlation, one-way ANOVA, and independent sample T-test. Then all the variables were entered in a multiple linear regression model with the OHRQoL as the dependent variable to control the effect of possible confounding factors. In all the analyses, an alpha level of 0.05 was regarded as the statistical significance.

### Ethical considerations

All the stages of the research were carried out under the Declaration of Helsinki. The Research Ethics Committee of Shiraz University of Medical Sciences reviewed and approved the study protocol (≠ IR.SUMS.REC.1394.S809). As the patients' information was recorded based on their code in the HIV/AIDS national software, they were unknown to the research team and their information was kept confidential. However, informed consent was obtained from all participants.

## Results

### Descriptive data

Of 219 participants, 149 (68.03%) were male individuals. The participants' age ranged from 22 to 73 years and their mean ± SD was 40.08 ± 7.94. They were diagnosed with HIV from 1996 to 2019; therefore, the duration of their disease ranged from 2 to 25 years with a mean ± SD of 7.66 ± 4.20 years. While 47.5% of participants had up to 8 children, 52.5% of them did not have any children (Tables 2, 3).

**Table 2:** Relationship between the participants' qualitative characteristics and OHRQoL was assessed using independent sample T-test and one-way ANOVA (N = 219)

Participants' characteristics	Measures	OHRQoL (Mean ± SD)	*P*-value*
*Sex, N (%)*			0.437
Woman	70 (31.97)	24.07 ± 6.39	
Man	149 (68.03)	24.78 ± 6.21	
*Marital status, N (%)*			0.495
Single	74 (33.80)	24.17 ± 5.83	
Married	84 (38.35)	25.19 ± 6.31	
Divorced or widowed	61 (27.85)	24.13 ± 6.73	
*Patient's education, N (%)*			0.847
0 < < 5-year education	89 (40.64)	24.26 ± 5.99	
6 ≤ < 12-year education	88 (40.18)	24.73 ± 6.47	
Having a diploma degree or university education	42 (19.18)	24.81 ± 6.52	
Patient's job, N (%)**			0.006
Unemployed or having a temporary job	118 (53.88)	24.18 ± 6.18^a^	
Having a permanent job	33 (15.07)	27.65 ± 6.20^b^	
Homemaker	68 (31.05)	23.64 ± 6.06^a^	
*Route of HIV transmission, N (%)*			0.569
Using share syringe by IV-drug abusers	128 (58.45)	25.05 ± 6.41	
Sexual contact outside the family	46 (21.00)	23.93 ± 5.93	
Transmission from her/his HIV^+^ spouse	37 (16.89)	23.67 ± 6.41	
Other routes	8 (3.66)	24.12 ± 5.25	
*Smoking cigarette or hookah (up to now), N (%)*			0.255
Yes	150 (68.49)	24.88 ± 6.31	
No	69 (31.51)	23.84 ± 6.15	
*Drinking alcohol (up to now), N (%)*			0.982
Yes	96 (43.84)	24.54 ± 6.47	
No	123 (56.16)	24.56 ± 6.13	
*Addiction to smoked illegal drugs (up to now), N (%)*			0.297
Yes	138 (63.00)	24.89 ± 6.27	
No	81 (37.00)	23.97 ± 6.26	
*Addiction to injected illegal drugs (up to now), N (%)*			0.409
Yes	119 (54.34)	24.87 ± 6.37	
No	100 (45.66)	24.17 ± 6.15	
*Having a previous history of imprisonment, N (%)*			0.218
Yes	130 (59.36)	24.98 ± 6.32	
No	89 (40.64)	23.92 ± 6.17	
*Edentulous Status, N (%)*			0.003
With edentulous	79 (36.07%)	26.21 ± 6.37	
Without edentulous	140 (63.93%)	23.61 ± 6.03	
*Wearing partial or complete denture, N (%)*			< 0.001
Yes	31 (13.70)	29.31 ± 5.57	
No	189 (86.30)	23.12 ± 5.74	

OHRQoL: Oral health-related quality of life

SD: Standard Deviation

*One way ANOVA was used for evaluation of the relationship between the patients' OHRQoL and their following characteristics: marital status, education, job, and route of HIV transmission. Other characteristics were assessed by using an independent sample T-test

**Tukey post hoc test was used for evaluation about the difference between subcategories of the patients' job. Different letters show statistically significant differences

**Table 3:** Relationship between the participants' quantitative characteristics and OHRQoL was assessed using Pearson correlation test (N = 219)

Participants' characteristics	Measures (Mean ± SD)	Univariate Analysis	
		Pearson Correlation (r)	*P*-value
Age	40.08 ± 7.94	0.170	0.012
Duration of disease	7.66 ± 4.20	0.176	0.009
Number of children	0.98 ± 1.40	0.023	0.733

OHRQoL: Oral health-related quality of life

SD: Standard Deviation

### The OHRQoL in patients infected with HIV

The mean scores determined for each question of OHRQoL varied from 2.40 ± 1.50 to 4.33 ± 1.06. The lowest and the highest scores were in psychosocial and pain categories, respectively. Although each patient's overall OHRQoL ranged from 11.00 to 35.00, the mean score of the OHRQoL was 24.55 ± 6.27 (Table 1).

### Factors affecting the patients' OHRQoL

In univariate analysis, we did not find a significant difference between the OHRQoL of women and men (*p* = 0.437). In addition, there was not a significant association between the participants' OHRQoL and many of their characteristics such as the route of HIV transmission (*p* = 0.569), cigarette or hookah smoking (*p* = 0.255), alcohol drinking (*p* = 0.982), and addiction to smoked and injected illegal drugs (*p* = 0.297 and *p* = 0.409, respectively). However, the analysis showed that the OHRQoL significantly improved by increasing the age and duration of the disease (*p* = 0.012 and *p* = 0.009, respectively). Moreover, the edentulous participants and those who were wearing partial or complete dentures had better OHRQoL (*p* = 0.003 and *p* < 0.001, respectively). Furthermore, the participants who had a permanent job had better OHRQoL (*p* = 0.006, Tables 2, 3). However, in multiple linear regression analysis, a significant difference was found between partial or complete denture wearing and non-denture wearing patients (*p* ≤ 0.001). No significant associations were found between the other variables and OHRQoL (Table 4).

**Table 4:** Multiple linear regression model was used to determine possible confounding factors. The participants' OHRQoL and characteristics were entered as dependent and independent variables, respectively (N = 219)

Variables	Sum of Squares	Mean Square	F	*P*-value
Sex	48.01	48.01	1.49	0.223
Marital status	58.77	29.38	0.91	0.403
Patient's education	46.70	23.35	0.73	0.485
Patient's job	165.64	82.82	2.57	0.079
Route of HIV transmission	82.28	27.43	0.85	0.467
Smoking cigarette or hookah	3.38	3.38	0.10	0.746
Drinking alcohol	11.46	11.46	0.35	0.551
Addiction to smoked illegal drugs	3.37	3.37	0.10	0.747
Addiction to injected illegal drugs	7.94	7.94	0.25	0.620
Having a previous history of imprisonment	5.39	5.39	0.17	0.683
Age	42.12	42.12	1.31	0.254
Duration of disease	126.94	126.94	3.94	0.051
Number of children	4.22	4.22	0.13	0.718
Edentulous status	59.44	59.44	1.85	0.176
Wearing a partial or complete denture	925.77	925.77	28.76	< 0.001

OHRQoL: Oral health-related quality of life

## Discussion

This cross-sectional study evaluated the OHRQoL and its risk factors in HIV-infected patients living in Shiraz. The patients stated that in the last 4 weeks, they had an average of 70% satisfaction with the OHRQoL items. The lowest and highest levels of satisfaction were from their concern about their mouth problems and the frequency of medication use, respectively. After controlling the confounding factors, we found that the patients who were wearing partial or complete dentures had better OHRQoL than those who were not.

In the present study, the HIV-infected patients obtained more than two-thirds of the OHRQoL scores. Various studies used different instruments for evaluating the OHRQoL in HIV-infected patients, which makes the comparisons difficult. However, all studies revealed that HIV-infected patients experienced a significantly worse OHRQoL for total and individual dimensions than the general population. This difference was evident even though the groups were similar in their socioeconomic backgrounds and health behavior risk factors [6, 9, 12, 13]. The OHRQoL in HIV-infected patients was also poorer than that of patients suffering from other diseases such as phobic dental anxiety, dentofacial deformity, and xerostomia [9]. Studies also revealed that the OHRQoL in HIV-infected patients has improved over time [10, 12]. This might be because of the positive changes that have happened in access to oral health care and dental coverage over time. It might be also due to the decrease in stigma and discrimination historically associated with HIV, and the greater willingness of dental practitioners to provide dental care for these patients [10]. A previous study also showed that the participants with oral symptoms had poorer OHRQoL than those without the symptoms [6]. Therefore, poor results of the OHRQoL in HIV-infected patients reported from several studies [8, 14, 15] supported the hypothesis that the patients had significant oral health needs. In addition, studies showed that HIV-infected patients living in the United States and Malaysia had much better OHRQoL than those in the present study [6, 10]. This finding highlights the importance of taking measures to improve oral health access for HIV-infected patients in Iran. The link between oral and general health is also well documented. Therefore, collaborations between medical and dental professionals are essential for the improvement of the delivery of oral health care services to HIV-infected patients [6].

We found the worst OHRQoL scores in the psychological category. The participants reported that in about half of the times in the last 4 weeks, they were worried or concerned about problems in their mouth. Mohamed and his coworkers also found that the most affected OHRQoL category in the HIV-infected adults in Malaysia was psychological discomfort. "Discomfort due to food getting stuck between the teeth or dentures" was the item within the psychological discomfort category that had the highest impact on the OHRQoL [6]. The present study also showed the best OHRQoL scores in the pain category and then the social category. Most of our participants reported that in the last 4 weeks, they did not use any medication to relieve pain or discomfort in their mouth. In contrast, Parish et al. [10] found the highest impact on OHRQoL for the items assessing the presence of painful aching in the mouth and discomfort while eating. Similar to the present study, Mohamed and his coworkers also showed that the lowest oral impact was found for social disability. In the study, no participant reported that the HIV infection often led to avoidance of going out [6]. In studies conducted by Adeniyi et al. [16] and Massarente et al. [17], oral symptoms were identified as one of the most affected categories of OHRQoL. These inconsistencies might be because of cultural differences and health promotion programs for HIV-infected patients in different countries, and the use of various instruments for evaluation of OHRQoL in different studies.

In the present research, similar to other studies [5, 8, 16, 17], no significant difference was found between female and male individuals' OHRQoL. On contrary, in Tomar et al. [15] study, women had worse OHRQoL than men. Although we found a significant positive correlation between the patients' age and OHRQoL, the correlation was not confirmed in the regression analysis. There is a controversy in this regard in the literature. Some studies reported that older age was associated with worse OHRQoL [8, 10]. In contrast, another study did not show a significant association between the patients' age and OHRQoL [16].

In the evaluation of socioeconomic factors, we found a significant association between the patients' job and OHRQoL in univariate analysis; however, it was not confirmed in the regression analysis. Other studies also showed worse OHRQoL for the unemployed patients [4, 7, 15], and those with lower education level [7] and lower personal monthly income [6, 7, 9, 10, 18]. Also, studies showed that the HIV-infected patients with adequate social support experienced better OHRQoL [10, 17]. For example, children who had their mother as the caretaker had better OHRQoL [17]. In addition, patients with low social support who had symptoms of anxiety, depression, and loneliness experienced lower OHRQoL than those without the symptoms [10].

In the present research, similar to other studies [8, 15], the patients' OHRQoL was not significantly associated with the duration of the disease. Also, there was no significant association between the patients' OHRQoL and their route of HIV transmission. In contrast, Coulter et al. [7] found that among patients infected with HIV, homosexual men had better OHRQoL than intravenous drug abusers. While a study explained that no HIV-related variables predicted OHRQoL [18], other investigations showed that the factors indicating the severity of HIV infection such as viral load [4, 17], CD4 cell counts [9], and AIDS diagnosis [7] were significantly associated with the patients' OHRQoL. Also, using or not using antiretroviral therapy [4] and the duration of the use [9] were found to be related to the patients' OHRQoL.

In the present study, the edentulous patients had significantly better OHRQoL than those who had at least one tooth in their mouth in univariate analysis. However, the study could not confirm this association after controlling the confounding factors in multiple regression analysis. In contrast, Mulligan et al. [9] showed that the total number of teeth was significantly related to OHRQoL, so that each additional tooth was associated with a 1% improvement in OHRQoL scores. In addition, in the present study, those who were wearing dentures had a significantly better OHRQoL than those who were not. Also, wearing denture was the only factor that had a significant association with the OHRQoL in multiple regression analysis. Similarly, Soares et al. [4] found an association between the need for dentures and worse OHRQoL. In contrast, Jeganathan et al. [18] found that the patients who were wearing a removable denture had a worse OHRQoL than those with natural dentition. Parish and coworkers showed that unmet dental needs had the strongest positive association with poor OHRQoL. They also revealed that the fulfillment of the needs could have a positive impact on the OHRQoL [10]. Rovaris et al. [8] study showed poor OHRQoL in HIV-infected children because of their high need for dental treatment. Therefore, although the care of HIV-infected patients significantly has improved recently, there are still important challenges regarding their management and treatment. This highlights the need for the establishment of multidisciplinary interventions in this regard.

## Recommendations

To improve the OHRQoL in HIV-infected patients, we recommend the policymakers design health programs to improve the patients' knowledge about the importance of oral health. Furthermore, for better health outcomes, dentists should be included in the team that manages the patients. Dentists should assess patients' OHRQoL during their management so that they can identify those who have significant oral health impacts [9]. It is recommended that public health programs should be held for better prevention and treatment of dental and oral problems in this vulnerable population. Regarding the high proportion of edentulous patients in this population, dentures need should be considered as an unmet need in the population. Therefore, to improve their oral health, authorities are recommended to provide facilities for helping the patients to become denture wearers. A study showed that there was a significant positive association between OHRQoL and general HRQoL [7]. Therefore, improving the patients' OHRQoL can improve their general HRQoL, leading to their health promotion.

## Limitations

Despite our great efforts to conduct a well-designed study, this study had some limitations. The most important one was the cross-sectional design of the study. Because of the design, it was impossible to be sure about the directionality of the noted associations. Although the participants of the present study were only those registered in a single VCT center, the center was the only referral center for the HIV-infected patients in Shiraz. According to guidelines in Iran, all HIV-infected patients should be referred to a VCT center. Therefore, the findings might be generalized to the HIV-infected patients in Shiraz. However, those patients that did not attend the center for follow-up care did not participate in the present study. Those without regular follow-up care were not treated with antiretroviral drugs. Furthermore, they might have relatively worse health status than those under the follow-up; therefore, the finding might be overestimated. Other limitations of the present study were recall and social desirability biases because our questionnaire was based on self-reported data and was administered by interviewers.

## Conclusions

Assessment of the OHRQoL in HIV-infected patients is important because it highlights their perceived needs and contributes to the planning of appropriate programs for improving their oral health. The present study showed that HIV-infected patients' OHRQoL was not optimal. Most patients were worried about the problems in their teeth and mouth. Therefore, we recommend active collaborations between medical and dental professionals to improve the delivery of oral health care services to HIV-infected patients. The present study also revealed that the OHRQoL was significantly better in the patients who were wearing partial or complete dentures. Therefore, policymakers can consider denture as an unmet need in these patients and provide facilities for helping the patients to become denture wearers.

## Data Availability

The datasets during the current study are not publicly available due to the confidentiality of the patient's data, but they will be available upon editorial reasonable request.

## Abbreviations

OHRQoL: Oral Health-Related Quality of Life
HIV: Human Immunodeficiency Virus
VCT: Voluntary Counseling and Testing
ANOVA: Analysis of variance
